# A Single Tertiary Center Experience in a South Asian Population: Does Tobacco Use Influence Bladder Cancer?

**DOI:** 10.7759/cureus.18734

**Published:** 2021-10-13

**Authors:** Nivash Selvaraj, Kunal Dholakia, Narasimhan Ragavan

**Affiliations:** 1 Urology, Apollo Hospitals, Chennai, IND

**Keywords:** bladder tumor, urinary bladder carcinoma, general population, smoking tobacco, bladder carcinoma

## Abstract

Introduction

Tobacco use, especially cigarette smoking, is a well-documented risk factor for cancer; however, its specific effect on bladder cancer has not been clearly defined. This study aimed to determine the association between tobacco use and bladder cancer in a South Asian population.

Materials and methods

We conducted a retrospective review of the medical records of 64 patients diagnosed with bladder tumors from February 2018 to March 2020. Patients included in the study were surveyed via a questionnaire regarding tobacco use. All patients received transurethral resection of the bladder tumor, and we analyzed histopathological and clinical outcomes.

Results

Our study population’s median age was 57 years, and the study included twice as many male patients as female patients. Most patients (n=45; 70%) reported not using tobacco products, and 19 patients (30%) reported tobacco use. Thirty-five of 45 nontobacco users (78%) had high-grade cancer, and 10 (22%) had low-grade cancer. Among the tobacco users, 10 (52%) had high-grade cancer, and nine (48%) had low-grade cancer.

Conclusions

According to our findings, a substantial cohort of bladder cancer patients is not tobacco users, and high-grade bladder cancer was more common to people who are not tobacco users. Other environmental factors play a key role in developing bladder cancer in our South Asian study population. Prevention efforts should focus on reducing bladder cancer risk factors.

## Introduction

Bladder cancer (BC) is potentially a deadly disease consisting of low-grade (LG) to high-grade (HG) tumors. Approximately 80% of patients have a non-muscle-invasive tumor, and 20% have a muscle-invasive tumor [1]. BC is associated with tobacco use, especially smoking, among other risk factors such as exposure to certain industrial chemicals, such as benzidine and beta-naphthylamine [2]. The literature offers conflicting evidence regarding the association between tobacco use intensity and BC [3-7]. Also, the treatment plan differs significantly between non-muscle-invasive BC and muscle-invasive BC. The present study aimed to evaluate the association between tobacco use and BC in a South Asian population.

## Materials and methods

We conducted a retrospective review of medical records of 64 patients diagnosed with BC from February 2018 to March 2020. All patients received a thorough clinical, hematological, and radiological workup before undergoing transurethral bladder tumor resection. We documented tobacco use, the type used (such as cigarette smoking, pipe smoking, and chewing tobacco), and duration via questionnaire. We reviewed patient demographics, including age and sex, and we reviewed clinical data, including presenting symptoms, preoperative tumor size (<3 cm or ≥3 cm), tumor grade (low or high), and histopathological data. Our center’s institutional review board approved the study design (ECR/36/Inst/TN/2020, Chennai).

Statistical analysis

Statistical analysis was performed using IBM SPSS Statistics for Windows, version 22.0 (Armonk, NY: IBM Corp.). We compared patient and tumor characteristics between tobacco and nontobacco groups patients using chi-square, Fisher’s exact test, and nonparametric Kruskal-Wallis test.

## Results

The median age of the patients who did not report tobacco use was 52 years [interquartile range (IQR), 48-71 years], and the median age of patients who reported tobacco use was 50 years (IQR, 46-70 years). The ratio of male patients to female patients in our study was 2:1 in both tobacco and nontobacco users. The most common symptom was macroscopic hematuria in both nontobacco users and tobacco users. Among those who reported using tobacco, the median duration of tobacco use was 30 years (IQR, 26-42 years). Table 1 presents the clinical characteristics of study participants.

**Table 1:** Clinical characteristics of bladder cancer patients IQR: interquartile range, LUTS: lower urinary tract symptoms, NA: not applicable.

Variable	Nontobacco Users (n=45)	Tobacco Users (n=19)
Age in years, median (IQR)	52 (48-71)	50 (46-70)
Sex n (%)		
Male	31 (69%)	13 (68%)
Female	14 (31%)	6 (32%)
Initial presentation, n (%)		
Macroscopic hematuria	29 (64%)	11 (59%)
Microscopic hematuria	9 (20%)	5 (26%)
LUTS	5 (11%)	2 (10%)
Others	2 (4%)	1 (5%)
Tobacco use, n (%)		
Smoking	NA	14 (74%)
Other forms	NA	5 (26%)
Years used, median (IQR)	NA	30 (26-42)

We found no significant relationship in tumor sizes between nontobacco users and tobacco users (p=0.58). However, a significant majority of patients with BC were nontobacco users (n=45; 70%), and only 19 patients with BC (30%) reported using tobacco. Also, among nontobacco users, 35 patients (78%) had HG cancer, and 10 (22%) had LG cancer. Among tobacco users, only 10 patients (52%) had HG cancer, and nine tobacco users (48%) had LG cancer. Urothelial carcinoma was common in both groups of patients (Table 2).

**Table 2:** Histopathological grading and tumor characteristics

Parameter	Nontobacco Users (n=45)	Tobacco Users (n=19)	P-value
Tumor size, n (%)			0.58
<3 cm	21 (47%)	11 (59%)	
≥3 cm	24 (53%)	8 (41%)	
Tumor grade, n (%)			
Low grade	10 (22%)	9 (48%)	0.07
High grade	35 (78%)	10 (52%)	
Nonmuscle invasive, n (%)	42 (93%)	17 (89%)	0.61
Muscle invasive, n (%)	3 (7%)	2 (11%)	0.02
Histology type			
Urothelial carcinoma	40	16	
Squamous cell carcinoma	3	2	
Adenocarcinoma	1	1	
Others	1	0	

## Discussion

BC is not common in the general population, inhibiting the establishment of a national-level screening program [8]. Identifying or defining those at high risk for BC would help a screening program become successful. Managing earlier stage BC yields excellent outcomes in prognosis and morbidity compared with advanced stage BC [9]. The most common presentation of BC is visible hematuria in many patients, as reported in our study. Hematuria makes the screening test unlikely to produce overdiagnosis [10]. Our study explored the association between tobacco use and BC in the South Asian population.

Smoking is a significant documented risk factor for cancer, and evidence suggests a significant association between smoking and tumor aggressiveness. In our study, however, BC was more prevalent among nontobacco users compared with that among tobacco users. This conflicts with Jiang et al., who reported a positive response between tumor aggressiveness and intensity of smoking [11].

A new molecular level BC subclassification was suggested by McConkey et al. regarding smoking intensity and BC [12]. Also, the Cancer Genome Atlas patients who had more aggressive tumor subtypes (e.g., basal-like carcinoma) began smoking earlier than patients with less aggressive tumor subtypes (e.g., luminal carcinoma) [13]. However, our results did not align with this trend.

In our study, HG cancers were more prevalent than LG cancers in either group, which conflicts with similar studies in the literature [14,15]. Therefore, HG tumors seem to be more common among the South Asian population than those among other populations. If so, it becomes more important to engage early screening programs in this population to diagnose BC early and allow patients the best chance for excellent outcomes.

A large majority of tobacco and nontobacco users had non-muscle-invasive cancer, and the most common histological finding was urothelial carcinoma, findings common to patient populations in other studies [16,17]. Therefore, cystoscopy examination is recommended for all patients presenting visible hematuria or other lower urinary tract symptoms, irrespective of tobacco use.

Our study had important limitations. This study occurred at a single tertiary referral center, and therefore, the patient population was more likely to have HG cancer due to the nature of the referral. Secondly, this was a retrospective study, and we did not investigate other risk factors like family history or environmental exposure, which can affect cancer development. The use of tobacco was patient reported, which could allow for inaccuracies.

## Conclusions

While tobacco use is a strong risk factor for BC, a substantial cohort of BC patients in our study reported that they did not use tobacco. Therefore, other environmental factors play a vital role in the development of BC. Programs for health education and prevention should continue to focus on reducing the incidence and prevalence of BC risk factors. Furthermore, HG cancers were much more common than LG cancers in this South Asian population. More extensive prospective studies should be conducted to verify this trend.

## References

[1] Siegel R, Ma J, Zou Z, Jemal A (2014). Cancer statistics, 2014. CA Cancer J Clin.

[2] Freedman ND, Silverman DT, Hollenbeck AR, Schatzkin A, Abnet CC (2011). Association between smoking and risk of bladder cancer among men and women. JAMA.

[3] Pietzak EJ, Malkowicz SB (2014). Does quantification of smoking history correlate with initial bladder tumor grade and stage?. Curr Urol Rep.

[4] Pietzak EJ, Mucksavage P, Guzzo TJ, Malkowicz SB (2015). Heavy cigarette smoking and aggressive bladder cancer at initial presentation. Urology.

[5] Rink M, Zabor EC, Furberg H (2013). Impact of smoking and smoking cessation on outcomes in bladder cancer patients treated with radical cystectomy. Eur Urol.

[6] Mitra AP, Castelao JE, Hawes D (2013). Combination of molecular alterations and smoking intensity predicts bladder cancer outcome: a report from the Los Angeles Cancer Surveillance Program. Cancer.

[7] Ajili F, Kourda N, Karay S, Darouiche A, Chebil M, Boubaker S (2013). Impact of smoking intensity on outcomes of patients with non muscle invasive bladder cancer treated by BCG immunotherapy. Ultrastruct Pathol.

[8] Krabbe LM, Svatek RS, Shariat SF, Messing E, Lotan Y (2015). Bladder cancer risk: use of the PLCO and NLST to identify a suitable screening cohort. Urol Oncol.

[9] Galsky MD, Domingo-Domenech J (2013). Advances in the management of muscle-invasive bladder cancer through risk prediction, risk communication, and novel treatment approaches. Clin Adv Hematol Oncol.

[10] Hollenbeck BK, Dunn RL, Ye Z (2010). Delays in diagnosis and bladder cancer mortality. Cancer.

[11] Jiang X, Castelao JE, Yuan JM (2012). Cigarette smoking and subtypes of bladder cancer. Int J Cancer.

[12] McConkey DJ, Choi W, Dinney CP (2014). New insights into subtypes of invasive bladder cancer: considerations of the clinician. Eur Urol.

[13] Sun X, Hoadley KA, Kim WY, Furberg H, Olshan AF, Troester MA (2017). Age at diagnosis, obesity, smoking, and molecular subtypes in muscle-invasive bladder cancer. Cancer Causes Control.

[14] Chamssuddin AK, Saadat SH, Deiri K, Zarzar MY, Abdouche N, Deeb O, Alia L (2013). Evaluation of grade and stage in patients with bladder cancer among smokers and non-smokers. Arab J Urol.

[15] Samaratunga H, Makarov DV, Epstein JI (2002). Comparison of WHO/ISUP and WHO classification of noninvasive papillary urothelial neoplasms for risk of progression. Urology.

[16] Brooks DR, Geller AC, Chang J, Miller DR (1992). Occupation, smoking, and the risk of high-grade invasive bladder cancer in Missouri. Am J Ind Med.

[17] Jensen OM, Wahrendorf J, Blettner M, Knudsen JB, Sørensen BL (1987). The Copenhagen case-control study of bladder cancer: role of smoking in invasive and non-invasive bladder tumours. J Epidemiol Community Health.

